# Effect of *Helicobacter pylori* eradication on the prognosis of older patients with chronic heart failure: a multicenter, retrospective cohort study

**DOI:** 10.3389/fmed.2026.1862816

**Published:** 2026-07-24

**Authors:** Yarong Chen, Pengfei Li, Yun Zhou, Li Zhao, Shengyi Yang, Xiaodong Zhang, Lumucao Bai, Jingwen Yuan, Mengru Wu, Xin Hu, Shixiong Liu

**Affiliations:** 1 The First School of Clinical Medicine of Lanzhou University, Lanzhou, Gansu, China; 2 Department of Geriatrics, The First Hospital of Lanzhou University, Lanzhou, Gansu, China; 3 Department of Medical Ultrasound, Affiliated Hospital of Gansu University of Traditional Chinese Medicine, Lanzhou, Gansu, China; 4 Intensive Care Unit, Tianshui Combine Traditional Chinese and Western Medicine Hospital, Tianshui, Gansu, China; 5 Intensive Care Unit, Zhangye Second People's Hospital, Zhangye, Gansu, China

**Keywords:** chronic heart failure, eradication, *Helicobacter pylori*, potential factors, prognosis

## Abstract

**Background:**

The current management of chronic heart failure (CHF) in older patients primarily focuses on traditional cardiovascular risk factors, with inadequate consideration of chronic infections such as *Helicobacter pylori (H. pylori)*. This study aimed to examine whether *H. pylori* eradication was associated with the prognosis of older patients with CHF and to identify potential factors.

**Methods:**

This multicenter, retrospective cohort study enrolled inpatients aged 55 years and older with Stage C heart failure and *H. pylori* infection from four hospitals (2022–2023). Participants were divided into the *H. pylori* eradication group and the non-eradication group. A 1:1 propensity score matching (PSM) was employed to construct a balanced cohort. The primary outcome was a composite endpoint of death due to heart failure and readmission for worsening heart failure. Kaplan-Meier and Log-Rank tests assessed survival differences, and Cox regression analysis identified potential factors. Subgroup and sensitivity analyses were also performed.

**Results:**

Among 191 patients, 50 (26.18%) experienced the composite endpoint during a median follow-up of 19.7 months. After 1:1 PSM, a well-balanced cohort of 156 patients (78 per group) was established, with 41 endpoint events (26.28%) recorded. Kaplan-Meier analysis showed higher event-free survival in the eradication group both before and after matching (*P* < 0.05). Multivariable Cox analysis revealed that *H. pylori* eradication was independently associated with a lower risk of the composite endpoint (HR: 0.27; 95% CI: 0.13–0.54, *P* < 0.001), while age ≥65 years and NYHA class III–IV were identified as independent risk factors. Subgroup analysis showed consistent associations across all subgroups (*P* for interaction >0.05).

**Conclusions:**

*H. pylori* eradication was independently associated with a lower risk of the composite endpoint of heart failure-related readmission and mortality in older patients (aged ≥55 years) with CHF. These findings suggest that *H. pylori* eradication deserves further investigation to determine its potential role in the comprehensive management of patients with heart failure and concurrent *H. pylori* infection. Prospective studies are needed to further evaluate the association and explore the underlying mechanisms.

## 1 Introduction

Chronic heart failure (CHF), the terminal stage of cardiovascular disease, is increasingly becoming a significant cause of mortality, readmission, and morbidity worldwide. It is estimated that over 60 million people suffer from heart failure globally. As the population ages, older adults have become the main population with heart failure, and there is an urgent need to reduce the increasing burden (1). Despite advances in treatment, the prognosis for older patients remains suboptimal, primarily due to the complexities of multimorbidity, adverse drug interactions, and a pervasive state of chronic low-grade inflammation (2). Therefore, identifying new intervention targets beyond traditional risk factors to enhance management is of great significance for improving the prognosis of older patients with CHF.

Currently, the clinical management of CHF in older patients mainly focuses on traditional risk factors, such as blood pressure, lipids, and glucose (3, 4), while screening and intervention for chronic infections are often overlooked. *Helicobacter pylori* (*H. pylori*) is a Gram-negative bacterium that causes chronic bacterial infection, with a global prevalence of 50%. It is not only closely associated with the development of gastrointestinal diseases such as chronic gastritis, peptic ulcers, and gastric mucosa-associated lymphoid tissue (MALT) lymphoma (5), but is also linked to the onset of various extra-gastrointestinal diseases (6, 7), such as cardiovascular disease (8), autoimmune diseases (9), neurological disorders (10, 11), diabetes (12, 13), asthma, and allergic diseases (14). Notably, the benefits of *H. pylori* eradication extend beyond the digestive system, with protective effects against diseases of the nervous, hematological, and endocrine systems having also been confirmed. For instance, *H. pylori* eradication can reduce the risk of ischemic stroke (11), increase platelet counts in patients with idiopathic thrombocytopenic purpura (9), and improve glycemic control in patients with type 2 diabetes by reducing insulin resistance (13). In the cardiovascular field, growing evidence suggests that *H. pylori* eradication can improve the prognosis of cardiovascular diseases such as coronary heart disease and myocardial infarction (15, 16). These benefits may result from the suppression of systemic chronic low-grade inflammation, thereby creating a more favorable microenvironment for cardiac function stability (17). However, although *H. pylori* eradication has demonstrated cardiovascular benefits in areas such as coronary heart disease, there are currently no studies reporting its effect on the prognosis of patients with CHF.

Based on this, we designed a multicenter, retrospective cohort study to investigate the association between *H. pylori* eradication and the prognosis of older patients (aged ≥55 years) with CHF and to identify potential factors.

## 2 Materials and methods

### 2.1 Study design and participants

This study is a multicenter, retrospective cohort study divided into the *H. pylori* eradication group (*H. pylori*-infected patients with successful eradication) and the non-eradication group (*H. pylori*-infected patients with failed eradication or those refusing eradication therapy). The study aimed to investigate the association between *H. pylori* eradication therapy and the prognosis of older patients with CHF and to identify potential prognostic factors.

The study included 191 patients aged 55 to 86 years who were hospitalized between January 2022 and December 2023 at the First Hospital of Lanzhou University, the Affiliated Hospital of Gansu University of Traditional Chinese Medicine, Zhangye Second People's Hospital, and Tianshui Combine Traditional Chinese and Western Medicine Hospital. These patients met the diagnostic criteria for Stage C heart failure and had concurrent *H. pylori* infection. The lower age limit of 55 years was selected to reflect the clinical spectrum of Stage C heart failure encountered in real-world practice, while the age cut-off of 65 years for subgroup and multivariate analyses was pre-specified based on the widely accepted international definition of older adults.

Patients enrolled in the study met the following inclusion criteria: (1) had a confirmed diagnosis of CHF (Stage C) and had been receiving standard treatment for more than a year; (2) were hospitalized at a participating center during the study period (January 2022–December 2023) and had a positive result on their first C^14^ urea breath test (C^14^-UBT); (3) possessed complete baseline clinical data and follow-up records for the primary endpoint events (death due to heart failure, readmission). Exclusion criteria were as follows: (1) severe missing baseline data (e.g., missing core cardiac function indicators or outcome data); (2) *H. pylori*-negative individuals; (3) history of *H. pylori* eradication; (4) acute coronary syndrome within 3 months prior to enrollment, or hospitalization due to severe acute cardiac decompensation (e.g., NT-proBNP >5,000 pg/ml); (5) concurrent uncontrolled systemic diseases: such as poorly controlled diabetes, thyroid dysfunction, active autoimmune diseases, severe hepatic or renal insufficiency (ALT/AST >3 times the upper limit of normal, eGFR < 30 ml/min), or a history of malignancy; (6) history of proton pump inhibitors, H_2_ receptor antagonists, or antibiotics within 2 months prior to enrollment; (7) history of cardiac surgery or major abdominal surgery.

### 2.2 Collection of clinical data

The general clinical data, including age, gender, combined atrial fibrillation, and medication history, as well as clinical laboratory data such as blood routine tests, blood biochemistry, body mass index (BMI), C^14^-UBT results, *H. pylori* subtype, and echocardiographic indicators—mainly including left ventricular ejection fraction (LVEF) and New York Heart Association (NYHA) functional class—were collected.

### 2.3 Diagnostic criteria and treatment for chronic heart failure

Diagnostic Criteria and Treatment Protocol: in accordance with the 2021 ESC Guidelines for the diagnosis and treatment of acute and chronic heart failure (18), patients met the criteria for Stage C heart failure (HF; Symptomatic HF: structural heart disease with current or previous symptoms of HF). Upon admission, all patients received standardized heart failure treatment in accordance with the guidelines, including angiotensin-converting enzyme inhibitors or angiotensin II receptor blockers (ACEI or ARB), angiotensin receptor neprilysin inhibitors (ARNI), beta-blockers, aldosterone receptor antagonists, sodium-glucose cotransporter two inhibitors (SGLT2i), etc. They were discharged after their symptoms had relieved.

### 2.4 Diagnosis, eradication, and efficacy assessment of *H. pylori* infection

*H. pylori* infection is diagnosed using the C^14^-UBT. Diagnostic criteria: C^14^-UBT count >100 dpm/mmol. If the test value is close to 100 dpm/mmol, repeat the test after a 2-week interval. *H. pylori* is classified based on antibodies as follows: type I is defined as positive for antibodies against cytotoxin-associated gene A (CagA) and/or vacuolating cytotoxin-associated gene A (VacA); Type II is defined as positive for antibodies against urease (Ure), with both anti-CagA and anti-VacA antibodies being negative.

Eradication treatment: for *H. pylori*-positive patients who meet the inclusion criteria, a 14-day quadruple therapy regimen containing bismuth was administered: esomeprazole magnesium enteric-coated tablets (20 mg each, taken twice daily before breakfast and dinner), bismuth potassium citrate (220 mg each, taken twice daily on an empty stomach, half an hour before breakfast and dinner), clarithromycin (0.25 g each, taken twice daily after breakfast and dinner), and tinidazole tablets (0.5 g each, taken twice daily after breakfast and dinner).

Efficacy assessment: a C^14^-UBT test was repeated 4 weeks after completion of eradication therapy, using the same testing method and criteria as before. Patients with negative results were deemed to have achieved successful eradication and were included in the *H. pylori* eradication group; those who remained positive were included in the *H. pylori* non-eradication group (including those with treatment failure and those who refused treatment).

### 2.5 Follow-up and outcomes

All patients continued to receive standardized heart failure treatment as needed after discharge, utilizing outpatient follow-up visits, proactive monitoring via the hospital's electronic medical record system, and standardized telephone follow-ups. Follow-ups were conducted monthly for the first 3 months after discharge, and every 3 months thereafter. The questionnaire was developed by the authors for the purpose of this study. Telephone follow-ups utilized a standardized questionnaire developed for this study (Supplementary File 1) covering current symptoms, physical condition, medication adherence, adverse reactions, and any hospitalizations or emergency department visits. Dietary habits and physical activity were recorded periodically; blood tests, including complete blood counts and liver and kidney function tests, were conducted regularly.

The primary outcomes are death due to heart failure and readmission due to worsening heart failure during the follow-up period. The follow-up period extends from discharge until death due to heart failure, the first readmission due to worsening heart failure symptoms, or the end of the study (December 31, 2023).

### 2.6 Statistical analysis

The measured data for normal distribution are presented as mean ± standard deviation (Mean ± SD), and an independent samples *t*-test is used to compare groups. The measured data for skewed distribution are reported as median (interquartile range) [M (IQR)], and the Mann–Whitney *U*-test is used to compare groups. Categorical data are shown as numbers and percentages, and the Pearson χ^2^ test or Fisher's exact test is used for comparisons between groups.

Propensity scores were estimated using a logistic regression model that included the following baseline covariates: age, sex, BMI, NYHA functional class, LVEF, *H. pylori* subtype, TC, TG, HDL-C, LDL-C, FBG, atrial fibrillation, and aspirin use. Patients in the eradication group were matched 1:1 to patients in the non-eradication group using nearest-neighbor matching with a caliper width of 0.02. Covariate balance before and after matching was assessed using standardized mean differences (SMD), with an absolute SMD < 0.1 considered indicative of adequate balance. Values between 0.1 and 0.2 were considered acceptable. For the propensity score-matched cohort, all covariates included in the propensity score model were simultaneously entered into the multivariable Cox regression model. This approach was adopted for two reasons. First, propensity score matching balanced the distribution of these covariates between groups, and simultaneous adjustment for all covariates provides a conservative estimate of the association by accounting for any residual imbalance. Second, all covariates were pre-specified based on clinical relevance and established risk factors for heart failure prognosis, rather than selected by statistical significance, to avoid model overfitting given the limited number of events in the matched cohort. Survival curves were plotted using the Kaplan-Meier method, and the Log-Rank test was used to assess differences in event-free survival between groups. Cox proportional hazards regression analysis was also performed to identify potential factors associated with the composite endpoint in patients with heart failure. Subgroup analyses were conducted to explore the association between eradication therapy and the composite endpoint in patients with different characteristics.

To further assess the potential influence of heterogeneity within the *H. pylori* non-eradication group, we conducted a sensitivity analysis. Patients in the *H. pylori* non-eradication group were further divided into the decline group and failure group, and the Kaplan-Meier method combined with the Log-Rank test was used to compare survival differences among the three groups (*H. pylori* eradication group, decline group, and failure group). Subsequently, using the *H. pylori* eradication group as the reference, the decline group and failure group were included as dummy variables in a multivariate Cox regression model. Meanwhile, we also compared the prognostic differences between the decline group and failure group, using the decline group as the reference.

Statistical analysis was performed using SPSS 27.0 and R version 4.5.1. A two-tailed *P* value < 0.05 was considered statistically significant.

## 3 Results

### 3.1 Baseline characteristics

**Figure 1:**
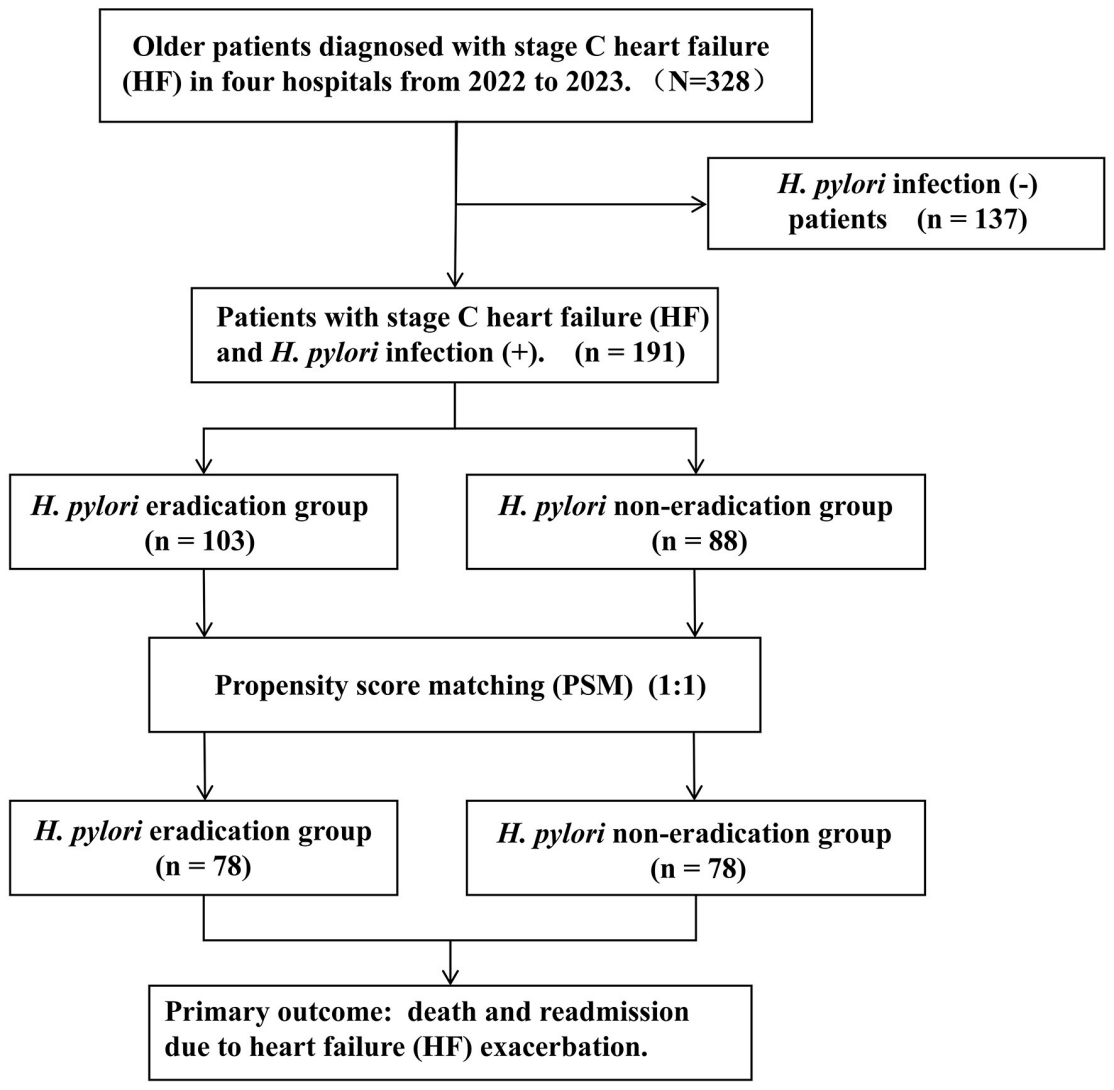
Flowchart of the study. PSM, propensity score matching; *H. pylori, Helicobacter pylori*.

From January 2022 to December 2023, 191 patients diagnosed with Stage C chronic heart failure (CHF) and concurrent *H. pylori* infection were enrolled based on strict inclusion and exclusion criteria (Figure 1). The age of the patients ranged from 55 to 86 years (median 67 years, IQR 63–71). Based on the success of *H. pylori* eradication, participants were divided into the *H. pylori* eradication group (*n* = 103) and the *H. pylori* non-eradication group (*n* = 88). The latter comprised 72 patients who declined eradication therapy and 16 who failed to achieve eradication post-treatment. The follow-up deadline was December 31, 2023. Table 1 shows the baseline characteristics of the enrolled patients. There were no statistically significant differences between the two groups in terms of age, gender, BMI, NYHA functional class, LVEF, *H. pylori* subtype, lipid profile, fasting blood glucose, combined atrial fibrillation, or aspirin use (all *P* > 0.05).

**Table 1:** Baseline characteristics in the *H. pylori* eradication group and the *H. pylori* non-eradication group before propensity score matching (*n* = 191).

Characteristics	Total (*n* = 191)	Group		SMD	*P*
		*H. pylori* eradication (*n* = 103)	*H. pylori* non-eradication (*n* = 88)		
Age [year, M (IQR)]	67 (63–71)	67 (63–71)	67 (63–71)	0.064	0.925^⋇^
Gender, *n* (%)				0.065	0.653
Male	103 (53.93)	54 (52.43)	49 (55.68)		
Female	88 (46.07)	49 (47.57)	39 (44.32)		
BMI [kg/m^2^, M (IQR)]	25.04 (23.15–27.12)	25.12 (23.19–27.38)	25.00 (22.94–26.64)	0.130	0.317^⋇^
NYHA functional class, *n* (%)				0.045	0.755
I–II	100 (52.36)	55 (53.40)	45 (51.14)		
III–IV	91 (47.64)	48 (46.60)	43 (48.86)		
LVEF, *n* (%)				0.205	0.161
< 40%	82 (42.93)	49 (47.57)	33 (37.50)		
≥40%	109 (57.07)	54 (52.43)	55 (62.50)		
*H. pylori*				0.016	0.911
I	131 (68.59)	71 (68.93)	60 (68.18)		
II	60 (31.41)	32 (31.07)	28 (31.82)		
TC [mmol/L, M (IQR)]	5.09 (4.52–5.85)	5.07 (4.52–5.85)	5.10 (4.52–5.91)	0.022	0.978^⋇^
TG [mmol/L, M (IQR)]	1.66 (1.19–2.46)	1.73 (1.21–2.28)	1.54 (1.14–2.50)	0.052	0.671^⋇^
HDL-C [mmol/L, mean ± SD]	1.520 ± 0.37	1.510 ± 0.36	1.540 ± 0.39	0.098	0.499^*^
LDL-C [mmol/L, M (IQR)]	2.49 (2.10–3.10)	2.49 (2.16–3.17)	2.51 (2.01–3.01)	0.086	0.525^⋇^
FBG [mmol/L, M (IQR)]	5.43 (5.09–5.99)	5.44 (5.09–5.99)	5.42 (5.10–6.11)	0.189	0.585^⋇^
Atrial fibrillation, *n* (%)	79 (41.36)	39 (37.86)	40 (45.45)	0.154	0.288
Aspirin, *n* (%)	145 (75.92)	78 (75.73)	67 (76.14)	0.010	0.948

^*^p-value calculated using independent samples Student's t-test.

^⋇^*p*-value calculated using Mann–Whitney U-test. The remaining *p*-value was calculated using the Pearson χ^2^ test or fisher's exact probability test.

M, median; IQR, interquartile range; SD, standard deviation; BMI, body mass index; NYHA, New York Heart Association; LVEF, left ventricular ejection fraction; H. pylori, Helicobacter pylori; TC, total cholesterol; TG, triglycerides; HDL-C, high-density lipoprotein cholesterol; LDL-C, low-density lipoprotein cholesterol; FBG, fasting blood glucose.

### 3.2 Primary outcome

**Figure 2:**
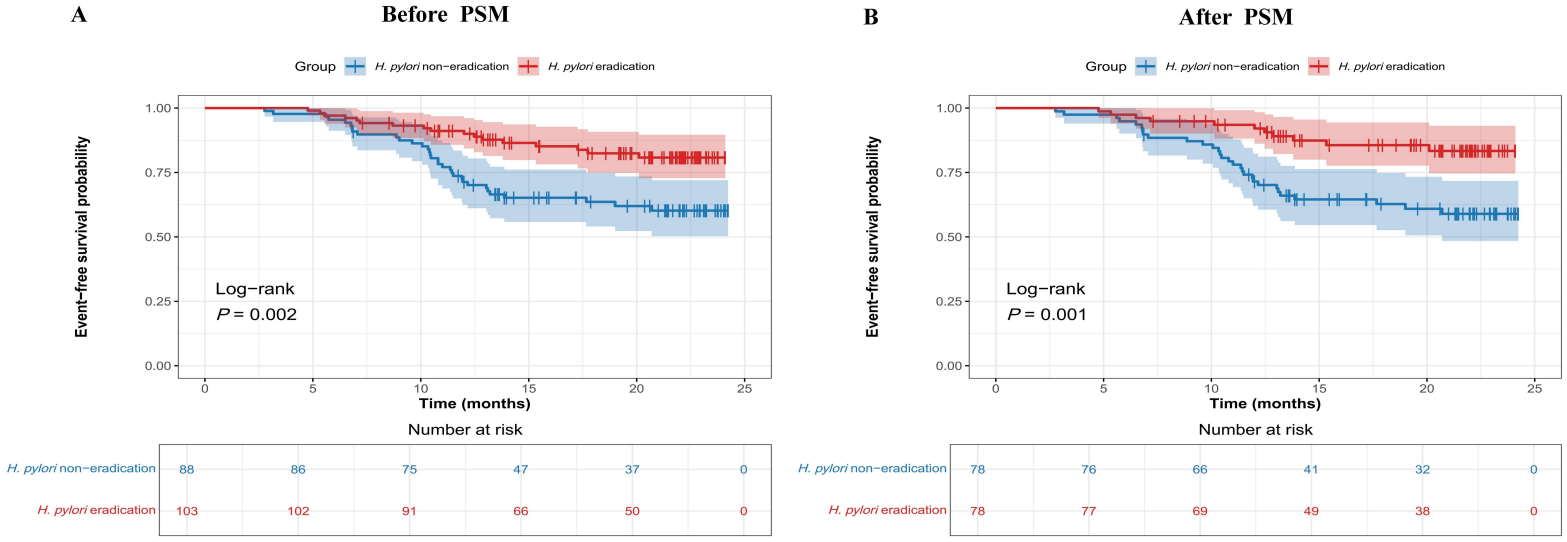
Event-free survival of older patients with chronic heart failure. (A) Event-free survival of patients in the *H. pylori* eradication group and the *H. pylori* non-eradication group; (B) Event-free survival of patients in the *H. pylori* eradication group and the *H. pylori* non-eradication group in the PSM cohort. Numbers at risk are shown below each curve. PSM, propensity score matching; *H. pylori, Helicobacter pylori*.

During a median follow-up period of 19.7 (12.3–22.8) months, 50 patients (26.18%) experienced the composite endpoint, with 17 (16.50%) in the *H. pylori* eradication group and 33 (37.50%) in the *H. pylori* non-eradication group. The difference in the incidence of the composite endpoint between the two groups was statistically significant (*P* = 0.001). Among the composite endpoint events, readmission occurred in 43 patients (22.51%); specifically, 16 cases (15.53%) in the *H. pylori* eradication group and 27 cases (30.68%) in the *H. pylori* non-eradication group, with a statistically significant difference (*P* = 0.012). Additionally, there were seven deaths (3.66%), with one case (0.97%) in the *H. pylori* eradication group and six cases (6.82%) in the *H. pylori* non-eradication group, a difference that was also statistically significant (*P* = 0.032; Table 2). Kaplan-Meier survival analysis showed that event-free survival was significantly higher in the *H. pylori* eradication group than in the *H. pylori* non-eradication group (*P* = 0.002; Figure 2A).

**Table 2:** Primary outcomes of the *H. pylori* eradication group and the *H. pylori* non-eradication group before propensity score matching.

Primary outcome	Total (*n* = 191)	Group		*P*
		*H. pylori* eradication (*n* = 103)	*H. pylori* non-eradication (*n* = 88)		
Composite endpoint	50 (26.18)	17 (16.50)	33 (37.50)	0.001
Readmission	43 (22.51)	16 (15.53)	27 (30.68)	0.012
Death	7 (3.66)	1 (0.97)	6 (6.82)	0.032

H. pylori, Helicobacter pylori.

### 3.3 Analysis of associated factors

Univariate and multivariate Cox regression analyses were performed to evaluate potential factors associated with the composite endpoint. Univariate analysis revealed that *H. pylori* eradication, NYHA functional class, and LVEF were associated with the occurrence of the composite endpoint (all *P* < 0.05). These statistically significant variables were included in a multivariate Cox proportional hazards regression model to assess their independent association with the composite endpoint. Multivariate analysis revealed that *H. pylori* eradication was independently associated with a lower risk of the composite endpoint (HR 0.35; 95% CI: 0.19–0.63, *P* < 0.001); NYHA functional class III–IV (HR 3.27; 95% CI: 1.76–6.09, *P* < 0.001) and LVEF < 40% (HR 2.45; 95% CI: 1.37–4.37, *P* = 0.002) were identified as independent risk factors for the composite endpoint (Table 3).

**Table 3:** Univariate and multivariate Cox regression analysis of factors associated with the composite endpoint for heart failure before propensity score matching.

Variables	Univariate		Multivariate	
	HR (95%CI)	*P*	HR (95%CI)	*P*
Age (≥65 years vs. < 65 years)	0.84 (0.47–1.49)	0.546		
Gender (male vs. female)	1.15 (0.66–2.00)	0.634		
BMI (≥23 kg/m^2^ vs. < 23 kg/m^2^)	1.18 (0.61–2.31)	0.622		
NYHA functional class (III–IV vs. I-II)	3.58 (1.93–6.64)	< 0.001	3.27 (1.76–6.09)	< 0.001
LVEF (< 40% vs. ≥40%)	2.23 (1.27–3.93)	0.005	2.45 (1.37–4.37)	0.002
*H. pylori* (II vs. I)	1.05 (0.58–1.90)	0.881		
TC (≥5.2 mmol/L vs. < 5.2 mmol/L)	0.87 (0.50–1.53)	0.634		
TG (≥1.7 mmol/L vs. < 1.7 mmol/L)	1.36 (0.78–2.37)	0.281		
HDL-C (< 1.0 mmol/L vs.≥1.0 mmol/L)	1.15 (0.41–3.20)	0.788		
LDL-C (≥3.4 mmol vs. < 3.4 mmol/L)	0.99 (0.44–2.20)	0.977		
FBG (≥6.1 mmol/L vs. < 6.1 mmol/L)	1.15 (0.60–2.20)	0.677		
Atrial fibrillation (yes vs. no)	0.75 (0.42–1.34)	0.333		
Aspirin (yes vs. no)	0.73 (0.40–1.33)	0.302		
*H. pylori* eradication (yes vs. no)	0.41 (0.23–0.73)	0.002	0.35 (0.19–0.63)	< 0.001

HR, hazard ratio; CI, confidence interval; BMI, body mass index; NYHA, New York Heart Association; LVEF, left ventricular ejection fraction; H. pylori, Helicobacter pylori; TC, total cholesterol; TG, triglycerides; HDL-C, high-density lipoprotein cholesterol; LDL-C, low-density lipoprotein cholesterol; FBG, fasting blood glucose.

### 3.4 Post-PSM analysis

To minimize the effect of bias in baseline confounding factors, the 1:1 PSM was performed to match the *H. pylori* eradication group with the *H. pylori* non-eradication group. A total of 156 patients were successfully matched, with 78 patients in each group. The absolute SMDs for all covariates were substantially reduced. Most covariates achieved SMDs below 0.1, with only BMI (SMD = 0.131) and *H. pylori* subtype (SMD = 0.136) falling within the acceptable range (0.1–0.2). Overall, the SMDs confirmed adequate balance between the two groups (Table 4).

**Table 4:** Baseline characteristics in the *H. pylori* eradication group and the *H. pylori* non-eradication group after propensity score matching (*n* = 156).

Characteristics	Total (*n* = 156)	Group		SMD	*P*
		*H. pylori* eradication (*n* = 78)	*H. pylori* non- eradication (*n* = 78)		
Age [year, M (IQR)]	67 (64–71)	67 (64–71)	67 (64–71)	0.040	0.629^⋇^
Gender, *n* (%)				0.026	0.872
Male	85 (54.49)	43 (55.13)	42 (53.85)		
Female	71 (45.51)	35 (44.87)	36 (46.15)		
BMI [kg/m^2^, mean ± SD]	24.802 ± 0.92	24.613 ± 0.22	25.002 ± 0.60	0.131	0.413^*^
NYHA functional class, *n* (%)				0.051	0.749
I–II	80 (51.28)	39 (50.00)	41 (52.56)		
III–IV	76 (48.72)	39 (50.00)	37 (47.44)		
LVEF, *n* (%)				< 0.001	1.000
< 40%	60 (38.46)	30 (38.46)	30 (38.46)		
≥40%	96 (61.54)	48 (61.54)	48 (61.54)		
*H. pylori*				0.136	0.398
I	103 (66.03)	49 (62.82)	54 (69.23)		
II	53 (33.97)	29 (37.18)	24 (30.77)		
TC [mmol/L, M (IQR)]	5.10 (4.56–5.82)	5.15 (4.60–5.85)	5.06 (4.55–5.84)	0.015	0.978^⋇^
TG [mmol/L, M (IQR)]	1.66 (1.15–2.47)	1.74 (1.18–2.26)	1.54 (1.13–2.53)	0.086	0.844^⋇^
HDL-C [mmol/L, Mean ± SD]	1.530 ± 0.37	1.540 ± 0.36	1.520 ± 0.38	0.047	0.768^*^
LDL-C [mmol/L, Mean ± SD]	2.560 ± 0.63	2.530 ± 0.61	2.590 ± 0.65	0.093	0.561^*^
FBG [mmol/L, M (IQR)]	5.42 (5.06–5.87)	5.43 (5.05–6.00)	5.42 (5.07–5.86)	0.088	0.743^⋇^
Atrial fibrillation, *n* (%)	67 (42.95)	33 (42.31)	34 (43.59)	0.026	0.872
Aspirin, *n* (%)	121 (77.56)	60 (76.92)	61 (78.21)	0.031	0.848

^*^p-value calculated using independent samples Student's t-test.

^⋇^*p*-value calculated using Mann–Whitney U-test.

The remaining *p*-value was calculated using the Pearson χ^2^ test or Fisher's Exact Probability Test.

M, median; IQR, interquartile range; SD, standard deviation; BMI, body mass index; NYHA, New York Heart Association; LVEF, left ventricular ejection fraction; H. pylori, Helicobacter pylori; TC, total cholesterol; TG, triglycerides; HDL-C, high-density lipoprotein cholesterol; LDL-C, low-density lipoprotein cholesterol; FBG, fasting blood glucose.

In the PSM cohort, a total of 41 patients (26.28%) experienced the composite endpoint, and the incidence of the composite endpoint was significantly higher in the *H. pylori* non-eradication group than in the *H. pylori* eradication group (38.46% vs. 14.10%, *P* < 0.001). Specifically, 11 cases (14.10%) of readmission occurred in the *H. pylori* eradication group, compared with 25 cases (32.05%) in the *H. pylori* non-eradication group; the difference between the two groups was statistically significant (*P* = 0.008). There were no deaths (0.00%) in the *H. pylori* eradication group and five deaths (6.41%) in the *H. pylori* non-eradication group, with a statistically significant difference between the two groups (*P* = 0.023; Table 5). Kaplan-Meier curves further showed that the event-free survival was significantly higher in the eradication group than in the non-eradication group (*P* = 0.001; Figure 2B).

**Table 5:** Primary outcomes of the *H. pylori* eradication group and the *H. pylori* non-eradication group after propensity score matching.

Primary outcome	Total (*n* = 156)	Group		*P*
		*H. pylori* eradication (*n* = 78)	*H. pylori* non- eradication (*n* = 78)	
Composite endpoint	41 (26.28)	11 (14.10)	30 (38.46)	< 0.001
Readmission	36 (23.08)	11 (14.10)	25 (32.05)	0.008
Death	5 (3.21)	0 (0.00)	5 (6.41)	0.023

H. pylori, Helicobacter pylori.

**Figure 3:**
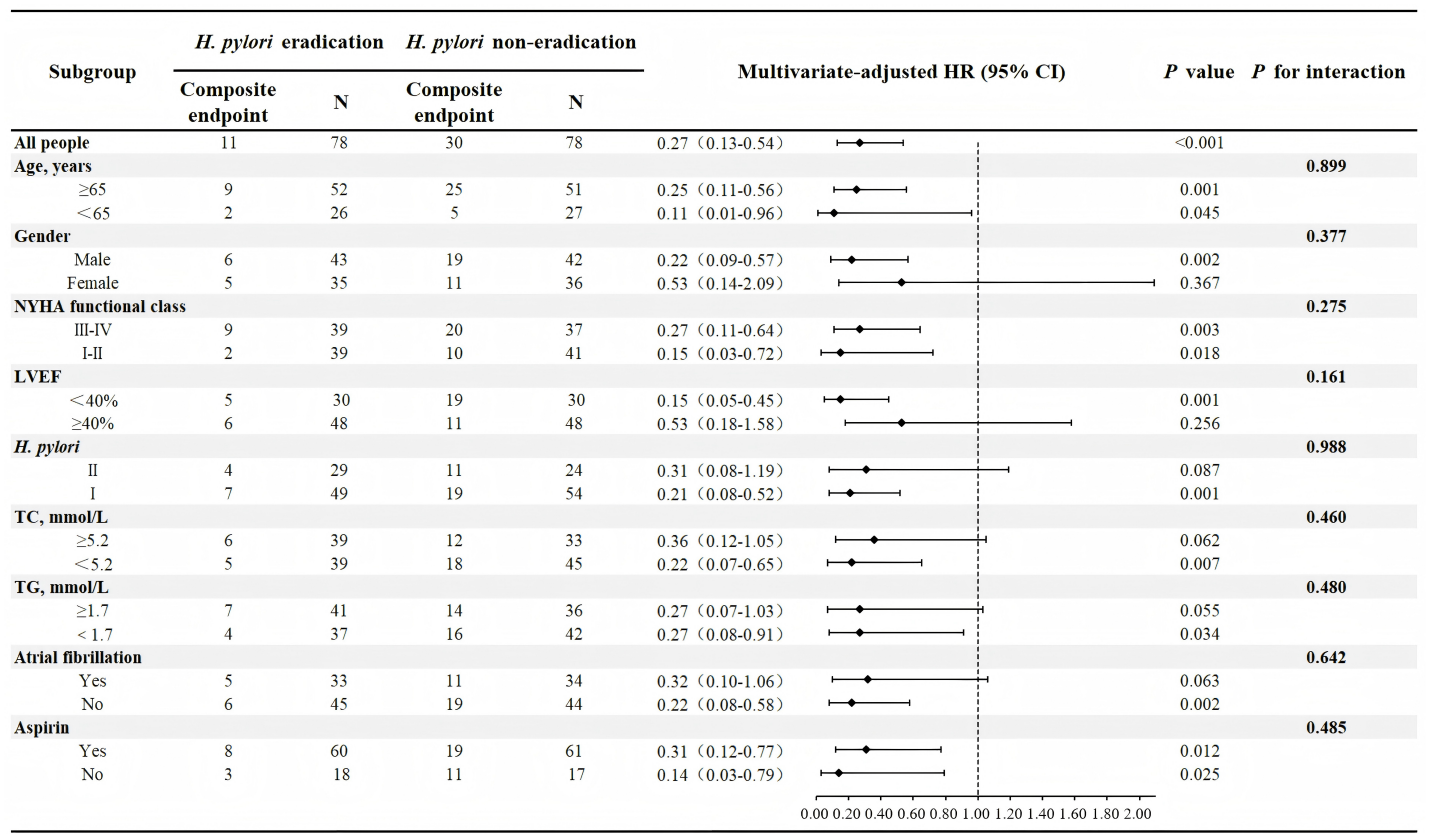
Subgroup analysis of the association between *H. pylori* eradication and the composite endpoint in the PSM cohort. Estimated effects were calculated using the Cox model adjusted by age, gender, BMI, NYHA functional class, LVEF, *H. pylori* subtype, TC, TG, HDL-C, LDL-C, FBG, atrial fibrillation, and aspirin use. HR, hazard ratio; CI, confidence interval; BMI, body mass index; NYHA, New York heart association; LVEF, left ventricular ejection fraction; *H. pylori, Helicobacter pylori*; TC, total cholesterol; TG, triglycerides; HDL-C, high-density lipoprotein cholesterol; LDL-C, low-density lipoprotein cholesterol; FBG, fasting blood glucose.

Multivariable Cox regression analyses of the PSM cohort showed that *H. pylori* eradication was independently associated with a lower risk of the composite endpoint (HR 0.27; 95% CI: 0.13–0.54, *P* < 0.001). In addition, age ≥65 years (HR 2.80; 95% CI: 1.12–6.99, *P* = 0.027) and NYHA functional class III–IV (HR 3.15; 95% CI: 1.48–6.68, *P* = 0.003) were identified as independent risk factors (Table 6). All covariates listed in Table 6 were simultaneously included in the multivariable Cox regression model. Interaction analyses were conducted for age, sex, NYHA functional class, LVEF, *H. pylori* subtype, lipid profile, presence of atrial fibrillation, and aspirin use. Subgroup analyses did not reveal significant heterogeneity in the association between *H. pylori* eradication and the composite endpoint across all subgroups (*P* for interaction > 0.05; Figure 3).

**Table 6:** Multivariate Cox regression analysis of factors associated with the composite endpoint for heart failure after propensity score matching.

Variables	HR (95%CI)	*P*
Age (≥65 years vs. < 65 years)	2.80 (1.12–6.99)	0.027
Gender (male vs. female)	1.21 (0.60–2.44)	0.588
BMI (≥23 kg/m^2^ vs. < 23 kg/m^2^)	1.77 (0.64–4.94)	0.275
NYHA functional class (III-IV vs. I-II)	3.15 (1.48–6.68)	0.003
LVEF (< 40% vs. ≥40%)	1.90 (0.94–3.85)	0.075
*H. pylori* (II vs. I)	1.63 (0.82–3.27)	0.165
TC (≥5.2 mmol/L vs. < 5.2 mmol/L)	0.78 (0.38–1.61)	0.505
TG (≥1.7 mmol/L vs. < 1.7 mmol/L)	1.32 (0.64–2.75)	0.452
HDL-C (< 1.0 mmol/L vs.≥1.0 mmol/L)	0.69 (0.15–3.26)	0.637
LDL-C (≥3.4 mmol vs. < 3.4 mmol/L)	0.73 (0.26–2.06)	0.555
FBG (≥6.1 mmol/L vs. < 6.1 mmol/L)	1.14 (0.54–2.44)	0.728
Atrial fibrillation (yes vs. no)	1.26 (0.62–2.57)	0.525
Aspirin (yes vs. no)	0.71 (0.33–1.52)	0.375
*H. pylori* eradication (yes vs. no)	0.27 (0.13–0.54)	< 0.001

HR, hazard ratio; CI, confidence interval; BMI, body mass index; NYHA, New York Heart Association; LVEF, left ventricular ejection fraction; H. pylori, Helicobacter pylori; TC, total cholesterol; TG, triglycerides; HDL-C, high-density lipoprotein cholesterol; LDL-C, low-density lipoprotein cholesterol; FBG, fasting blood glucose.

### 3.5 Sensitivity analysis

Before PSM, the *H. pylori* non-eradication group was further divided into the decline group (*n* = 72) and the failure group (*n* = 16). A comparison of baseline data across the three groups showed that all characteristics were evenly distributed among the three groups (all *P* > 0.05), suggesting comparable baseline characteristics across these subgroups (Supplementary Table S1). Survival analysis revealed significant differences in event-free survival among the *H. pylori* eradication group, decline group, and failure group (*P* = 0.004; Supplementary Figure S1). Using the *H. pylori* eradication group as the reference, multivariate Cox regression analysis showed that the failure group (HR = 2.98; 95% CI: 1.18–7.52, *P* = 0.021) and the decline group (HR = 2.53; 95% CI: 1.31–4.90, *P* = 0.006) had significantly elevated risks of the composite endpoint (Supplementary Figure S2). Meanwhile, when using the decline group as the reference, the difference between this group and the failure group was not statistically significant (HR = 1.18; 95% CI: 0.48–2.89, *P* = 0.722; Supplementary Figure S3).

## 4 Discussion

This multicenter retrospective cohort study found that *H. pylori* eradication was independently associated with a lower risk of the composite endpoint of heart failure-related readmission and mortality in older patients (aged ≥55 years) with CHF. Additionally, multivariate Cox regression analysis showed that age and NYHA functional class were independent risk factors for CHF prognosis.

Currently, research on the relationship between *H. pylori* infection and cardiovascular disease (CVD) remains inconsistent. Some studies indicated that *H. pylori* infection may be associated with an increased risk of CVD (8, 19). For example, a cross-sectional study found that *H. pylori* infection is associated with increased risk of dyslipidemia, and *H. pylori* eradication may prevent the occurrence of cardiovascular disease by reducing the occurrence of dyslipidemia (20). Another study showed that chronic *H. pylori* infection expressing CagA was associated with elevated levels of BNP and IL-6 in the blood of patients with non-ST elevation acute coronary artery disease (ACAD), and that IL-6 levels were linked to short and long-term mortality in heart failure (21). Meanwhile, a Mendelian randomization study also suggested that the effect of *H. pylori* infection on coronary heart disease (CHD) incidence may be mediated by BMI, and that eradication or prevention of *H. pylori* infection may provide indirect clinical benefits for CHD patients (22). However, some studies have shown that chronic *H. pylori* infection is not associated with CVD incidence (23, 24). For instance, Christodoulou DK et al. (23) suggested that there was no association of *H. pylori* infection with CVD and eradication of *H. pylori* to prevent CVD was not warranted.

Against this backdrop, our study provides preliminary evidence suggesting that *H. pylori* eradication is independently associated with favorable prognosis in older patients with CHF and concurrent *H. pylori* infection. To our knowledge, this is the first multicenter study to examine this association in this population. However, previous studies regarding the cardiovascular associations of *H. pylori* eradication have been inconsistent. A Korean cohort study confirmed that both the direct effect of *H. pylori* eradication and the indirect effect of lifestyle modifications contribute to the prevention of cardiovascular disease (25). Similarly, long-term follow-up from another large-scale cohort study indicated that *H. pylori* eradication can prevent the onset of coronary heart disease, with this effect varying by age and gender (26). However, although a study involving 4,269 participants found that while *H. pylori* eradication improved lipid profiles, it did not significantly reduce the risk of cardiovascular disease (27). Also, another cohort study based on the NHIRD showed that *H. pylori* eradication significantly reduced the composite endpoint of CHD and death, as well as the cumulative incidence of CHD in individuals under 65 years. However, its effect on those aged 65 or older was unclear (15). This study adds to the limited evidence for this age group, showing that in older patients with CHF and *H. pylori* infection, successful *H. pylori* eradication was associated with a lower risk of composite endpoint events (HR 0.27, 95% CI 0.13–0.54), further supporting this association.

Subgroup analyses did not reveal significant heterogeneity across the examined subgroups. However, given the limited sample size and number of events, these subgroup findings should be interpreted as exploratory. At the same time, sensitivity analysis further supported the robustness of the results. It indicates that patients with unsuccessful *H. pylori* eradication, whether due to eradication failure or refusal of treatment, were associated with a poorer prognosis, and further emphasizes the importance of successful eradication. It is worth noting that, in the sensitivity analysis, the sample size in the failure group was small, which may have limited the statistical power to compare differences in prognosis between this group and the decline group. The finding that no statistically significant difference was observed between the two groups should be interpreted with caution. These findings suggest that *H. pylori* eradication warrants further investigation as a potential consideration in the management of older patients with heart failure and concurrent *H. pylori* infection.

The potential mechanism by which *H. pylori* eradication may be associated with favorable prognosis in older patients with CHF could be related to the suppression of systemic chronic low-grade inflammation. Heart failure itself provides the pathological basis for chronic inflammation; specifically, elevated central venous pressure causes splanchnic congestion (28, 29), subsequently resulting in intestinal wall edema, mucosal barrier damage, and dysbiosis (30–32). This cascade facilitates the translocation of endotoxins (e.g., lipopolysaccharide [LPS]) into the bloodstream, activating the Toll-like receptor 4 (TLR4) signaling pathway (33) and ultimately elevating levels of inflammatory cytokines such as TNF-α and IL-6 (34, 35). In this context, *H. pylori* infection may act as an additional inflammatory stimulus, potentially exacerbating the pre-existing inflammatory state. Previous studies have reported that *H. pylori* infection can upregulate pro-inflammatory cytokines such as TNF-α, IL-6, and CRP, whereas *H. pylori* eradication therapy can reduce levels of IL-1β, IL-8, and TNF-α (36, 37). Long-term exposure to these inflammatory cytokines triggers chronic inflammatory cascades and lipid metabolism dysregulation, which not only directly damage cardiomyocytes but also promote myocardial fibrosis and ventricular remodeling (38, 39), thereby accelerating the progression of heart failure. *H. pylori* eradication effectively may remove this additional inflammatory stimulus. It may reduce the systemic inflammatory burden by reversing cytokine changes and improving the lipid profile, thereby potentially creating a more favorable microenvironment for cardiac function stabilization.

Notably, this study also further validated the classic determinants of heart failure prognosis. We found that older age and higher NYHA functional class are strong predictors of composite endpoint events in patients with heart failure, consistent with the findings of several large-scale studies (40–42). In contrast, gender was not significantly associated with the composite endpoint in our cohort. Although some prior studies have suggested sex-related differences in CHF outcomes (43–47), this negative finding may be attributable to our specific older population with concurrent *H. pylori* infection and the relatively limited sample size. Future sex-stratified analyses with larger cohorts are warranted to further clarify this relationship.

This study has several limitations. First, as a retrospective observational study, it cannot establish causality, and residual confounding cannot be completely ruled out. Although propensity score matching (PSM) was used to adjust for measured covariates, unmeasured confounders such as socioeconomic status, healthcare-seeking behavior, medication adherence, frailty, cognitive status, and overall health status may still simultaneously influence both the willingness to undergo eradication and the prognosis of heart failure. Second, the follow-up period was limited (median 19.7 months), and eradication status was assessed only once 4 weeks after treatment, without regular review, so long-term effects, sustainability of benefit, and the impact of *H. pylori* reinfection could not be assessed. Additionally, the retrospective design did not systematically collect data on adherence, tolerability and adverse events, limiting a comprehensive assessment of the benefit-risk ratio. Third, reliance on telephone follow-ups and electronic records carries theoretical risks of information bias and incomplete event capture. However, if such bias exists, it usually tends to non-differential misclassification, which would make the effect estimate tend to zero and would not change the main conclusions of this study. Fourth, this study included only *H. pylori*-positive patients; it did not include a negative control group nor patients who had previously undergone eradication therapy. Therefore, it addresses the association between eradication of current infection and prognosis, rather than whether *H. pylori* infection itself is an independent prognostic factor for heart failure. Fifth, the non-eradication group included patients who refused eradication therapy (*n* = 72) and those in whom eradication failed (*n* = 16), representing fundamentally heterogeneous subgroups. Although we conducted subgroup comparisons in sensitivity analyses, the sample size in the failure group (16 cases) was too small, severely limiting statistical power and making it impossible to reliably assess differences between groups. This limitation may have led to a rough estimate of the effect, but it does not affect the main conclusions. Sixth, to control for confounding and ensure treatment safety, this study employed relatively strict inclusion and exclusion criteria. While this enhanced internal validity, it also resulted in a relatively selected subgroup of older patients with heart failure. Therefore, the results have limited generalizability to patients with severe multisystem comorbidities, those in different stages of heart failure, or those with stable outpatient heart failure. Seventh, the study population was drawn from a region in northwestern China with a high prevalence of *H. pylori*. Therefore, the results may not be applicable to older adults in areas with low prevalence or to different ethnic groups. In conclusion, the results of this study are still exploratory. Future large-scale prospective randomized controlled trials are necessary to further validate the study's conclusions and elucidate the underlying mechanisms.

## 5 Conclusions

This multicenter, retrospective cohort study found that *H. pylori* eradication was independently associated with a lower risk of the composite endpoint of heart failure-related readmission and mortality in older patients (aged ≥55 years) with CHF. We also found that age ≥65 years and NYHA functional class III–IV were independently associated with a higher risk of the composite endpoint. Given the observational design, residual confounding cannot be excluded. Nonetheless, *H. pylori* eradication deserves further investigation to determine its potential role in the comprehensive management of patients with heart failure and concurrent *H. pylori* infection. Prospective studies are needed to further evaluate the association and explore the underlying mechanisms.

## Data Availability

The original contributions presented in the study are included in the article/Supplementary material, further inquiries can be directed to the corresponding author.
