# Supplementary material for: Effect of Helicobacter pylori eradication on the prognosis of older patients with chronic heart failure: a multicenter, retrospective cohort study
Source: Front Med (Lausanne). 2026 Jul 24;13:1862816. doi: 10.3389/fmed.2026.1862816 (PMC13447156; doi:10.3389/fmed.2026.1862816)
Supplement: Supplementary file 1 [file Table_1.docx]

***Supplementary Material***

Number of supplementary figures: 3

Number of supplementary tables: 1

**Supplementary Table S1** Baseline characteristics in the eradication group, decline group, and failure group before propensity score matching (n = 191).

**Supplementary Figure S1** Event-free survival of three groups before propensity score matching.

**Supplementary Figure S2** Forest plot of the association between *H. pylori* eradication status and the composite endpoint before propensity score matching.

**Supplementary Figure S3** Forest plot comparing the risk of the composite endpoint between the decline group and the failure group before propensity score matching.

**Supplementary Table S1** Baseline characteristics in the eradication group, decline group, and failure group before propensity score matching (n = 191).

| **Characteristics** | ***H. pylori* eradication**  **(n=103**) | ***H. pylori* non-eradication**  **(n=88**) | | ***P*** |
| --- | --- | --- | --- | --- |
|  |  | **Decline group**  **(n=72**) | **Failure group**  **(n=16**) |  |
| Age [year, M(IQR)] | 67(63-71) | 67(63-71) | 67(65-70) | 0.935^※^ |
| Gender, n (%) |  |  |  | 0.462 |
| Male | 54(52.43) | 38(52.78) | 11(68.75) |  |
| Female | 49(47.57) | 34(47.22) | 5(31.25) |  |
| BMI [ kg/m^2^, M(IQR)] | 25.12(23.19-27.38) | 24.77(23.21-26.63) | 25.31(22.43-27.18) | 0.602^※^ |
| NYHA functional class, n (%) |  |  |  | 0.948 |
| I-II | 55(53.40) | 37(51.39) | 8(50.00) |  |
| III-IV | 48(46.60) | 35(48.61) | 8(50.00) |  |
| LVEF, n (%) |  |  |  | 0.201 |
| ＜40% | 49(47.57) | 25(34.72) | 8(50.00) |  |
| ≥40% | 54(52.43) | 47(65.28) | 8(50.00) |  |
| *H. pylori* |  |  |  | 0.458 |
| I | 71(68.93) | 47(65.28) | 13(81.25) |  |
| II | 32(31.07) | 25(34.72) | 3(18.75) |  |
| TC [ mmol/L, M(IQR)] | 5.07(4.52-5.85) | 5.12(4.56-5.80) | 4.89(4.37-6.03) | 0.957^※^ |
| TG [mmol/L, M(IQR)] | 1.73(1.21-2.28) | 1.54(1.16-2.49) | 1.50(1.02-2.78) | 0.909^※^ |
| HDL-C [ mmol/L, Mean ± SD] | 1.51±0.36 | 1.57±0.41 | 1.43±0.26 | 0.331* |
| LDL-C [ mmol/L, M(IQR)] | 2.49(2.16-3.17) | 2.51(2.01-3.01) | 2.53(1.96-3.18) | 0.812^※^ |
| FBG [ mmol/L, M(IQR)] | 5.44(5.09-5.99) | 5.42(5.13-6.11) | 5.47(4.96-6.05) | 0.840^※^ |
| Atrial fibrillation, n (%) | 39(37.86) | 34(47.22) | 6(37.50) | 0.441 |
| Aspirin, n (%) | 78(75.73) | 55(76.39) | 12(75.00) | 0.991 |

Note: *: p-value calculated using one-way analysis of variance (ANOVA). ^※^: p-value calculated using Kruskal‑Wallis H test. The remaining p-value calculated using Pearson χ^2^ test or Fisher's Exact Probability Test. Abbreviations: M, median; IQR, interquartile range; SD, standard deviation; BMI, body mass index; NYHA, New York Heart Association; LVEF, left ventricular ejection fraction; *H. pylori*, *Helicobacter pylori*; TC, total cholesterol; TG, **triglycerides; HDL-C, high-density lipoprotein cholesterol; LDL-C,** low**-density lipoprotein cholesterol; FBG**, **fasting blood glucose.**

**Supplementary Figure S1** Event-free survival of three groups before propensity score matching. Note: Abbreviations: *H. pylori*, *Helicobacter pylori*.

**Supplementary Figure S2** Forest plot of the association between *H. pylori* eradication status and the composite endpoint before propensity score matching. Estimated effects were calculated using the Cox model adjusted by age, gender, BMI, NYHA functional class, LVEF, *H. pylori* subtype, TC, TG, HDL-C, LDL-C, FBG, atrial fibrillation, and aspirin use. HR, hazard ratio; CI, confidence interval; BMI, body mass index; NYHA, New York Heart Association; LVEF, left ventricular ejection fraction; *H. pylori*, *Helicobacter pylori*; TC, total cholesterol; TG, **triglycerides; HDL-C**, **high-density lipoprotein cholesterol; LDL-C**, low**-density lipoprotein cholesterol; FBG**, **fasting blood glucose.**

**Supplementary Figure S3** Forest plot comparing the risk of the composite endpoint between the decline group and the failure group before propensity score matching. Estimated effects were calculated using the Cox model adjusted by age, gender, BMI, NYHA functional class, LVEF, *H. pylori* subtype, TC, TG, HDL-C, LDL-C, FBG, atrial fibrillation, and aspirin use. HR, hazard ratio; CI, confidence interval; BMI, body mass index; NYHA, New York Heart Association; LVEF, left ventricular ejection fraction; *H. pylori*, *Helicobacter pylori*; TC, total cholesterol; TG, **triglycerides; HDL-C**, **high-density lipoprotein cholesterol; LDL-C**, low**-density lipoprotein cholesterol; FBG, fasting blood glucose.**
