# Supplementary material for: Effect of Helicobacter pylori eradication on the prognosis of older patients with chronic heart failure: a multicenter, retrospective cohort study
Source: Front Med (Lausanne). 2026 Jul 24;13:1862816. doi: 10.3389/fmed.2026.1862816 (PMC13447156; doi:10.3389/fmed.2026.1862816)
Supplement: Supplementary file 2 [file Table_2.docx]

**Supplementary File 1: Standardized Follow-up Questionnaire**

**Title: Follow-up questionnaire for the study: “**Effect of Helicobacter pylori eradication on the prognosis of older patients with chronic heart failure**”**

Background and Purpose**: This questionnaire was specifically developed for the present multicenter, retrospective cohort study. It was administered via standardized telephone interviews to systematically and consistently collect data on clinical status,** medication **adherence, adverse reactions, and the occurrence of pre-defined endpoint events (heart failure** readmission **and death) among enrolled older patients with chronic heart failure.**

Instructions: This questionnaire was administered by trained research staff during scheduled telephone follow-ups (monthly for the first 3 months after discharge, then every 3 months thereafter). Responses were recorded based on patient self-report.

**Section A: Patient identification and contact information**

Study ID: _______________

Date of follow-up: ______

Follow-up time point: □ 1 Month □ 2 Months □ 3 Months □ 6 Months □ 9 Months □ 12 Months □ Other: ______

**Section B: Current clinical status and symptoms**

**1.General condition since last follow-up:**

Since your last contact, how have you been feeling regarding your heart condition?

- Much better

□ Somewhat better

□ About the same

□ Somewhat worse

□ Much worse

**2.Symptom checklist (Since last follow-up)**

Since your last follow-up, have you experienced any of the following symptoms?

| Symptom | Yes | No | If yes, please describe severity |
| --- | --- | --- | --- |
|  |  |  | (mild/moderate/severe) |
| Chest tightness or chest pain | □ | □ | _______________ |
| Shortness of breath (dyspnea) at rest or on exertion | □ | □ | _______________ |
| Orthopnea (difficulty breathing when lying flat) | □ | □ | _______________ |
| Paroxysmal nocturnal dyspnea (waking up breathless at night) | □ | □ | _______________ |
| Peripheral edema (swelling of ankles, feet, or legs) | □ | □ | _______________ |
| Fatigue or reduced exercise tolerance | □ | □ | _______________ |
| Palpitations | □ | □ | _______________ |
| Dizziness or syncope | □ | □ | _______________ |

**3.NYHA functional class (based on activity limitation)**

How much does your heart condition limit your physical activity?

□ **Class I:** No limitation. Ordinary physical activity does not cause symptoms.
**□ Class II****:** Slight limitation. Comfortable at rest, but ordinary physical activity results in symptoms (e.g., shortness of breath, fatigue).
**□ Class III:** Marked limitation. Comfortable at rest, but less than ordinary activity causes symptoms.
**□ Class IV:** Symptoms at rest. Unable to carry on any physical activity without discomfort.

**4.Overall health change (open-ended)**

Have you noticed any other changes in your health since your last follow-up?

Response: ____________________________________________________________

**Section C: Medication adherence and changes**

**1. Adherence to heart failure medications**

Have you been taking your heart failure medications exactly as prescribed?”

- Yes, as prescribed (no missed doses)
- Mostly, but missed 1–2 doses in the past month
- Partially, missed several doses
- No, stopped taking one or more medications
- Not applicable / unable to answer

If non-adherent, ask reason(s) (check all that apply):

□ Side effects

□ Forgot

□ Cost

- Felt medication was not needed
- Other (specify): _______________

**2. Current heart failure medications (check if still taking)**

□ ACEI / ARB

□ ARNI

□ Beta-blocker

□ Aldosterone receptor antagonist

□ SGLT2i

□ Diuretic

□ Other: _______________

**3. Changes in medication regimen since last follow-up**

Have there been any changes to your heart failure medications since the last follow-up (e.g., dose adjustment, new medication, discontinuation by doctor)?

- No changes
- Yes, there have been changes (please specify): ____________________________________________

**Section D: Adverse reactions**

Since your last follow-up, have you experienced any new or worsening side effects that you think might be related to your medications?

- No
- Yes (please describe)

Adverse reaction	Onset date	Action taken (e.g., reduced dose, stopped, contacted doctor)

_____________________	______________	________________________________________________

Commonly reported examples: dry cough, hypotension, bradycardia, hyperkalemia, dizziness, gastrointestinal upset, rash.

_____________________________________

**Section E:** **Hospitalizations or Emergency Department Visits (critical for endpoint ascertainment)**

**1. Hospitalization since last follow-up**

Since your last follow-up, have you been admitted to a hospital for any reason?

- No
- Yes → If yes:

Date of hospitalization: __________________

Primary reason: __________________

Was the primary reason worsening heart failure symptoms?

- Yes
- No

Duration of stay (days): __________________

Name of hospital: __________________

**2. Emergency department visits since last follow-up**

Since your last follow-up, have you visited an emergency department for any reason?

- No
- Yes → If yes:

Date of visit: __________________

Primary reason: __________________

Was the primary reason worsening heart failure symptoms?

- Yes
- No

For heart failure-related readmission, record the primary symptoms that led to the visit:

________________________________________________

**Section F: Lifestyle factors**

Have you made any notable changes to your diet (e.g., salt/fluid restriction) or physical activity/exercise routine since your last follow-up?

- No significant changes
- Yes (please briefly describe): ____________________________________________

Telephone follow-up questionnaire. ACEI: angiotensin-converting enzyme inhibitor; ARB: angiotensin receptor blocker; ARNI: angiotensin receptor neprilysin inhibitor; SGLT2i: sodium-glucose cotransporter 2 inhibitor.
